# Regulation of alternative splicing of Bcl-x by BC200 contributes to breast cancer pathogenesis

**DOI:** 10.1038/cddis.2016.168

**Published:** 2016-06-09

**Authors:** R Singh, S C Gupta, W-X Peng, N Zhou, R Pochampally, A Atfi, K Watabe, Z Lu, Y-Y Mo

**Affiliations:** 1 Department of Biochemistry, Cancer Institute, University of Mississippi Medical Center, Jackson, MS, USA; 2 Department of Cell Biology, School of Medicine, Jiangsu University, Zhenjiang, China; 3 System Biosciences, Mountain View, CA, USA; 4 Cancer Biology, Wake Forest School of Medicine, Bermuda Run, NC, USA; 5 Department of Endocrinology, PLA General Hospital, Beijing, China; 6 Department of Pharmacology/Toxicology and Cancer Institute, University of Mississippi Medical Center, Jackson, MS, USA

## Abstract

BC200 is a long non-coding RNA (lncRNA) that has been implicated in the regulation of protein synthesis, yet whether dysregulation of BC200 contributes to the pathogenesis of human diseases remains elusive. In this study, we show that BC200 is upregulated in breast cancer; among breast tumor specimens there is a higher level of BC200 in estrogen receptor (ER) positive than in ER-negative tumors. Further experiments show that activation of estrogen signaling induces expression of BC200. To determine the significance of ER-regulated BC200 expression, we knockout (KO) BC200 by CRISPR/Cas9. BC200 KO suppresses tumor cell growth *in vitro* and *in vivo* by expression of the pro-apoptotic Bcl-xS isoform. Mechanistically, BC200 contains a 17-nucleotide sequence complementary to Bcl-x pre-mRNA, which may facilitate its binding to Bcl-x pre-mRNA and recruitment of heterogeneous nuclear ribonucleoprotein (hnRNP) A2/B1, a known splicing factor. Consequently, hnRNP A2/B1 interferes with association of Bcl-x pre-mRNA with the Bcl-xS-promoting factor Sam68, leading to a blockade of Bcl-xS expression. Together, these results suggest that BC200 plays an oncogenic role in breast cancer. Thus, BC200 may serve as a prognostic marker and possible target for attenuating deregulated cell proliferation in estrogen-dependent breast cancer.

Long non-coding RNAs (lncRNAs) provide a new perspective to the central dogma of gene expression, where DNA is also transcribed into RNAs that do not code for protein, in addition to protein-coding mRNAs.^1^ Among the total human transcripts, about 20 000 are protein-coding genes, accounting for <2% of the human genome. The rest of them are non-coding RNAs, including microRNAs and lncRNAs. Since there is a lack of a satisfactory method for the classification of lncRNAs, they are arbitrarily considered to be longer than ~200 nucleotides and can go up to over 100 kb.^2^ LncRNAs are involved in the regulation of various biological processes, such as genome imprinting, gene regulation, chromatin organization and alternative splicing.^3^ Thus, they can serve as master gene regulators through various mechanisms,^4^ and their deregulation may lead to a variety of diseases such as diabetes, neurodegenerative disorders, cardiovascular disease and cancer.^5^ For example, a number of lncRNAs have been implicated in different aspects of tumorigenesis such as tumor cell growth and proliferation, invasion and metastasis, and thus, they can function as oncogenes or tumor suppressor genes.^6^

BC200, also called BCYRN1 (brain cytoplasmic RNA 1), is a 200-nucleotide in length, and is transcribed by RNA polymerase III.^7^ BC200 has been shown to act as a translational modulator, regulating synaptodendritic protein synthesis in neurons.^8^ It is prevalently expressed in the nervous system, but not in non-neural organs such as colon, heart, kidney, liver, spleen or skeletal muscle.^9, 10^ However, BC200 is also detected in a number of tumors such as breast, cervix, esophagus, lung, ovary, parotid and tongue, whereas it is not detectable in corresponding normal tissues^10^ or in benign tumors such as fibroadenomas.^11^

Despite upregulation of BC200 in various cancers, the role of BC200 in cancer development and progression, as well as the underlying mechanism of BC200-mediated gene regulation in cancer remains poorly understood. In the present study, we report that BC200 is transcriptionally induced by estrogen in breast cancer cells, and it prevents apoptosis by modulating alternative splicing of a member of the Bcl-2 family, Bcl-x.^12^

## Results

### Upregulation of BC200 in breast tumor specimens

**Figure 1:**
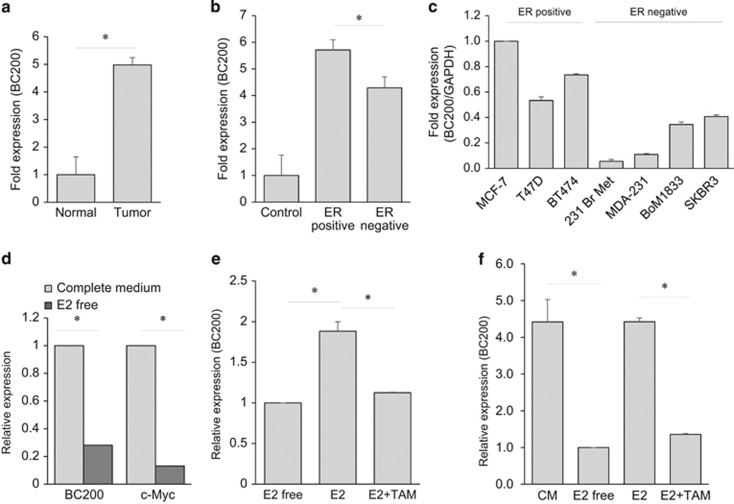
Aberrant expression of lncRNA BC200 in breast cancer. (**a**) Average fold change for BC200 in breast cancer tissue scan array (OriGene) analyzed by qPCR shows a significant upregulation in breast tumors (*n*=43) as compared with normal tissue (*n*=5). (**b**) ER-positive breast cancer samples (*n*=22) have a higher expression than ER-negative samples (*n*=10). (**c**) Detection of BC200 by qRT-PCR in ER^+^ and ER^−^ breast cancer cell lines. (**d** and **e**) Effect of estradiol (E2) and tamoxifen (TAM) on BC200 expression. T47D cells were grown in E2-free medium and treated with 1 nM E2 alone or in combination with 5 *μ*M TAM for 18 h. (**f**) Same as (**d**) and (**e**), but in MCF-7 cell. Values for (**d**) and (**e**) are means±S.E. (*n*=3); *P<0.05

To investigate expression of BC200 in tumor specimens, we used a breast cancer tissue cDNA array from OriGene (http://www.origene.com/) consisting of 43 breast cancer and 5 normal breast tissue samples. As determined by qRT-PCR analysis, BC200 was highly expressed in breast cancer as compared with normal tissues (Figure 1a). Of great importance, the status of estrogen receptor (ER) impacted expression of BC200 among tumor specimens. For example, ER-positive breast cancer tumors had a higher level of BC200 than those ER-negative tumors (Figure 1b and Supplementary Figure S1A). This trend was also seen in breast cancer cell lines. For example, the level of BC200 in ER-positive cells, such as MCF-7 and T47D, was higher than in ER-negative cells such as MDA-MB-231 (Figure 1c). This finding prompted us to further investigate the role of estrogen (E2) in regulation of BC200 expression.

### Transcriptional regulation of BC200 by estrogen

In initial qRT-PCR experiments using MCF-7 and T47D cells, we found that E2 deprivation caused a significant decrease in BC200 expression (Figure 1d). Similar to BC200, c-Myc, a known gene that is regulated by E2,^13^ was also suppressed by E2 deprivation (Figure 1d). Interestingly, exposure of the cells that were previously grown in E2-free medium to E2 increased the level of BC200 by a 1.8-fold in T47D cells (Figure 1e). Similar results were also seen in MCF-7 cells, reinforcing the notion that activation of E2 signaling induces expression of BC200. To determine whether this effect is mediated through ER, we treated these cells with E2 in the presence or absence of tamoxifen (TAM). As shown in Figure 1e and f, TAM suppressed the E2-induced BC200 expression in both T47D and MCF-7 cells, further suggesting BC200 as an estrogen-regulated target lncRNA.

**Figure 2:**
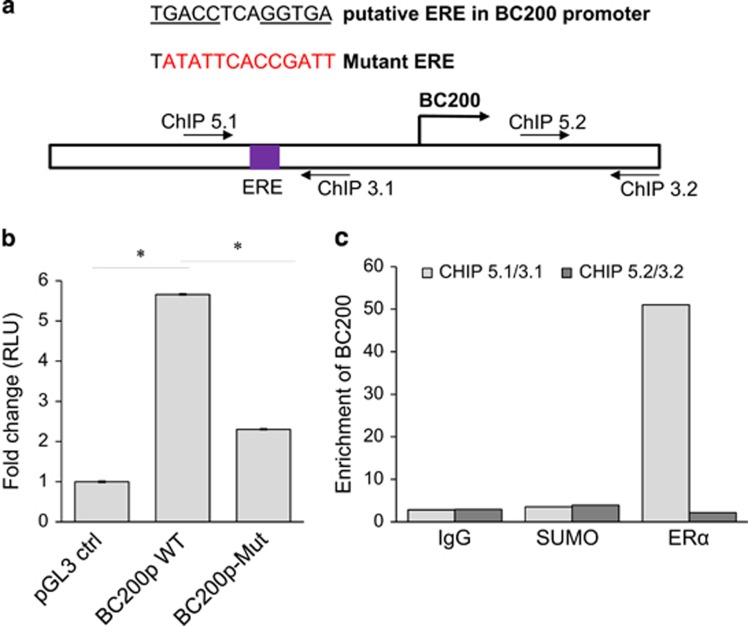
BC200 is transcriptionally regulated in an estrogen-dependent manner. (**a**) BC200 promoter carries a functional estrogen response element (ERE). A schematic description of ERE in the putative promoter of BC200 and locations of primers used for ChIP assay. The 2-kb region of BC200 promoter with wild-type ERE or mutant ERE (highlighted in red) was cloned into pGL3-basic vector. (**b**) Luciferase assays for reporters carrying a 2-kb region of BC200 promoter with wild-type ERE or mutant ERE. MCF-7 cells were grown in a 12-well plate in E2-free medium and co-transfected with 400 ng of ERE-pGL3 constructs along with 2 ng of pRL-SV40 renilla luciferase construct as an internal transfection control. At 24 h post-transfection, cells were treated with 1 nM E2, incubated for an additional 18 h and then harvested for luciferase assays. Renilla luciferase was used for normalization. (**c**) ChIP assay to confirm ER*α* binding to ERE in BC200 promotor. BC200-ChIP-5.1 and BC200-ChIP-3.1 cover ERE site; BC200-ChIP-5.2 and BC200-ChIP-3.2 are derived from downstream of BC200, serving as a negative control for PCR. Immunoglobulin G and non-specific antibody (anti-SUMO) were used as negative controls for immunoprecipitation. Values are means±S.E. (*n*=3); *P<0.05

Scanning of the putative BC200 promoter revealed the presence of potential estrogen response elements (EREs) within a 2-kb region upstream of BC200 transcription start site (Supplementary Figure S2A), particularly the site (**TGACC**TCA**GGTGA**) located at −585-bp region (Figure 2a). Thus, we cloned this 2 kb DNA fragment into a luciferase reporter pGL3-basic vector. Luciferase assays revealed over a fivefold increase in promoter activity on treatment with E2 (Figure 2b). Mutagenesis analysis indicated that this site plays an important role in E2-mediated BC200 promoter activity (Figure 2b). Furthermore, chromatin immunoprecipitation (ChIP) assay confirmed the binding of ER to this ERE-containing region (Figure 2c; Supplementary Figure S2B). Together, these results suggest that ER interacts with the putative ERE in the BC200 promoter to induce its transcription.

### BC200 is critical to cell proliferation and survival

**Figure 3:**
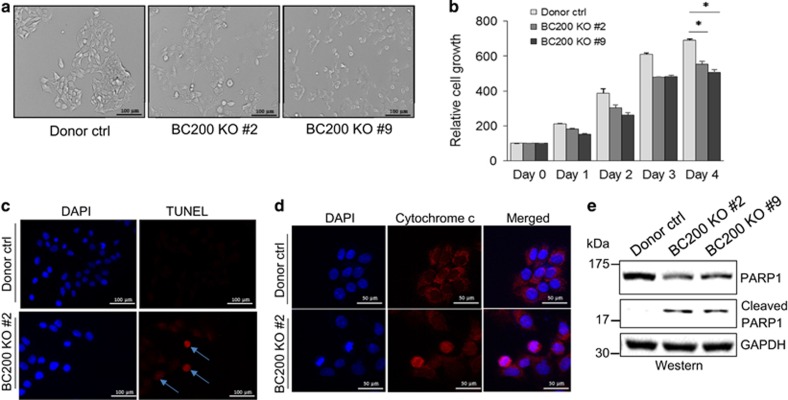
Knockout of BC200 inhibits cell growth and promotes apoptosis. (**a**) Morphological changes of MCF-7 cells after BC200 KO as compared with vector control cells. (**b**) Cell proliferation was determined by MTT assay. (**c**) TUNEL assay reveals more positive signals in BC200 KO cells than in control cells. The nucleus was stained with Hoechst 33342. (**d**) Immunostaining with cytochrome *c* antibody detects cytochrome *c* release from mitochondria to cytosol in BC200 KO cells. (**e**) Western blot shows PARP1 cleavage in BC200 KO cells, but not in control cells. Values are means±S.E. (*n*=3); *P<0.05

Since a number of E2-regulated genes, such as c-Myc,^14^ play an important role in breast tumorigenesis, next we sought to determine the significance of BC200 in breast cancer. Thus, we knocked out BC200 using CRISPR/Cas9 system^15^ since RNAi was not effective for BC200. Accordingly, we deleted BC200 in MCF-7 cells using the CRISPR/Cas9 system combined with a dual gRNA (guide RNA) approach we recently developed.^16^ These two gRNAs were located at the 5′ and 3′ extremities of the BC200 gene (Supplementary Figure S3A), enabling us to delete the entire gene. A challenge for gene knockout (KO) in cancer cell lines is the polyploidy nature. For example, MCF-7 cells are hypotetraploid. In this case, we may need to simultaneously KO four copies of a given gene to obtain a complete KO clone. To facilitate screening of KO clones, we generated a donor vector carrying left and right arms for homologous recombination (HR).^16^ KO clones were initially identified by genomic PCR (Supplementary Figure S3B), and then further confirmed by qRT-PCR analysis (Supplementary Figure S3C). This approach identified several complete KO clones, and we selected two of them (KO#2 and KO#9) for the following experiments. As shown in Figure 3a, BC200 KO caused significant morphologic changes and alterations in cell viability. MTT assay confirmed that BC200 KO suppressed the cell growth significantly as compared with vector control cells (Figure 3b).

Terminal deoxynucleotidyl transferase dUTP nick end labeling (TUNEL) assay detected a high rate of apoptosis in BC200 KO cells (Figure 3c). Furthermore, immunofluorescence analysis with the cytochrome *c* antibody demonstrated a mitochondria leakage in BC200 KO cells, whereas vector control cells revealed distinct speckles within the mitochondria (Figure 3d). We also found increased cleavage of PARP1 in BC200 KO cells relative to vector control cells (Figure 3e), providing further evidence that BC200 plays an anti-apoptotic role.

### BC200 KO suppresses tumor growth in the xenograft mouse model

**Figure 4:**
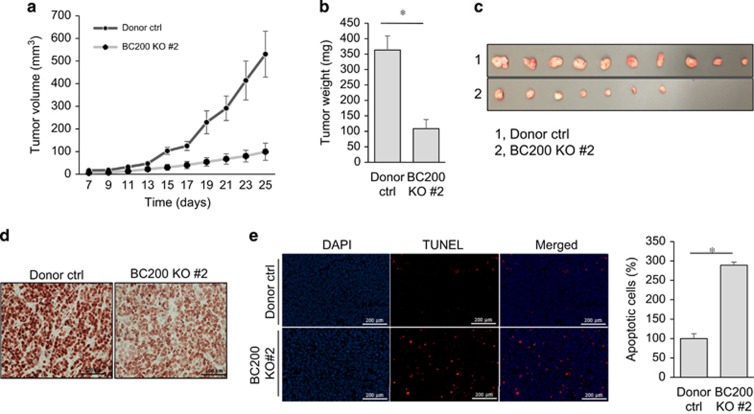
Suppression of tumor growth *in vivo* by BC200 KO. (**a**) MCF-7 control cells or BC200 KO cells were injected into mammary fat pad of female nude mice. Tumor growth was measured every other day, 7 days after the injection. (**b**) Average weight of the excised tumors for vector control or BC200 KO. (**c**) Actual tumor sizes. (**d**) A decreased Ki-67 level in BC200 KO tumors, as detected by IHC. (**e**) Increased apoptosis in tumors derived from vector control or BC200 KO cells, as determined by TUNEL assay (left). The graph on the right shows average percentage of tumor cells (calculated by randomly choosing five different fields under microscope) undergoing apoptosis in control and BC200 KO group, where control was normalized as 100%. Values are means±S.E. (*n*=3); *P<0.05

Furthermore, orthotopic injection of MCF-7 cells carrying vector control or BC200 KO into female nude mice revealed that BC200 KO significantly reduced tumor growth (Figure 4a), resulting in a reduction of tumor weight (Figures 4b and c). As expected, we detected little expression of BC200 in BC200 KO tumors (Supplementary Figure S4A); there was a weak Ki-67 signal for BC200 KO tumors as compared with vector control (Figure 4d). TUNEL assays revealed more apoptosis in BC200 KO tumors as compared with control tumors (Figure 4e). Together, these results further support the important role of BC200 in breast tumor cell growth and proliferation.

### BC200 KO increases the level of pro-apoptotic Bcl-xS isoform

**Figure 5:**
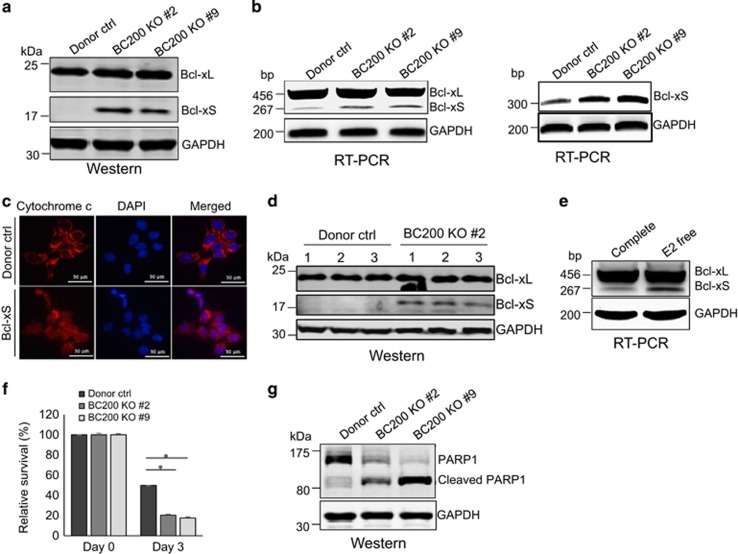
BC200 KO induces Bcl-xS to promote apoptosis. (**a**) Detection of the pro-apoptotic Bcl-xS isoform in BC200 KO cells as compared with vector control by western blot. (**b**) Detection of Bcl-x isoforms by RT-PCR in vector control and BC200 KO cells. Left panel, this set of primers were able to amplify both Bcl-xL and Bcl-xS; right panel, this set of primers were specific to Bcl-xS. (**c**) Ectopic expression of Bcl-xS promotes cytochrome *c* release in MCF-7 cells. (**d**) Detection of Bcl-xS in tumors derived from BC200 KO cells by western blot. (**e**) E2 deprivation increases Bcl-xS, as detected by RT-PCR. (**f**) BC200 KO sensitizes MCF-7 cells to anti-cancer drug doxorubicin (doxo). The cells were treated with 0.5 *μ*g/ml doxo for up to 3 days. Relative cell survival was determined by MTT assay. (**g**) BC200 KO increases PARP1 cleavage after doxo treatment, as detected by western blot. Values are means±S.E. (*n*=3); *P<0.05

To determine how BC200 KO induces apoptosis, we analyzed the expression of apoptosis regulating proteins such as Bcl-2 and Bcl-x. There was no difference for Bcl-2 and Bax between control and BC200 KO cells although the levels of Bim were slightly increased in BC200 KO cells (Supplementary Figure S4B). Of interest, we detected the short form of Bcl-x (Bcl-xS) in the BC200 KO cells, but this band was undetectable in vector control by western blot (Figure 5a). It is well-known that the long form of Bcl-x (Bcl-xL) plays an anti-apoptotic role, whereas Bcl-xS is pro-apoptotic in nature.^17^ Selection of the proximal 5′ splice site in exon 2 promotes the anti-apoptotic long variant, Bcl-xL, whereas selection of the distal 5′ splice site promotes the pro-apoptotic short variant, Bcl-xS. To determine whether the Bcl-xS protein is generated through alternative splicing of Bcl-x, we performed RT-PCR and found that Bcl-xS mRNA was increased in BC200 KO cells as compared with vector control (Figure 5b) although we also detected a low level of Bcl-xS mRNA in control cells. Bcl-xS has been shown to induce apoptosis in different cancer cell lines.^18, 19, 20^ Congruent with these studies, ectopic expression of Bcl-xS (Supplementary Figure S5A) caused release of cytochrome *c* from mitochondria to cytosol (Figure 5c), similar to what was seen in BC200 KO cells. Finally, we detected Bcl-xS in tumors derived from BC200 KO cells, but not from control tumors (Figure 5d). These results suggest that BC200 KO promotes apoptosis at least in part through expression of pro-apoptotic Bcl-xS.

By inducing BC200 transcription, E2 could conceivably regulate Bcl-x mRNA alternative splicing, causing inhibition of Bcl-xS expression. Indeed, E2 deprivation increased expression of Bcl-xS mRNA (Figure 5e), supporting its role in the BC200-mediated regulation of Bcl-x alternative splicing. Upregulation of Bcl-xS expression has been shown to enhance the sensitivity of cancer cells to chemotherapeutics agents.^18^ Thus, we examined the effect of BC200 KO in response to doxorubicin (doxo) treatment. MTT assay showed a significant cell inhibition in BC200 KO cells as compared with vector control (Figure 5f). Moreover, western blot detected a marked increase in PARP1 cleavage in BC200 KO cells as compared with vector control on treatment with doxo (Figure 5g). These results suggest that BC200 KO can sensitize MCF-7 cells to anti-cancer agents.

### BC200 specifically regulates Bcl-x alternative splicing through interaction with the splicing factor hnRNP A2/B1

**Figure 6:**
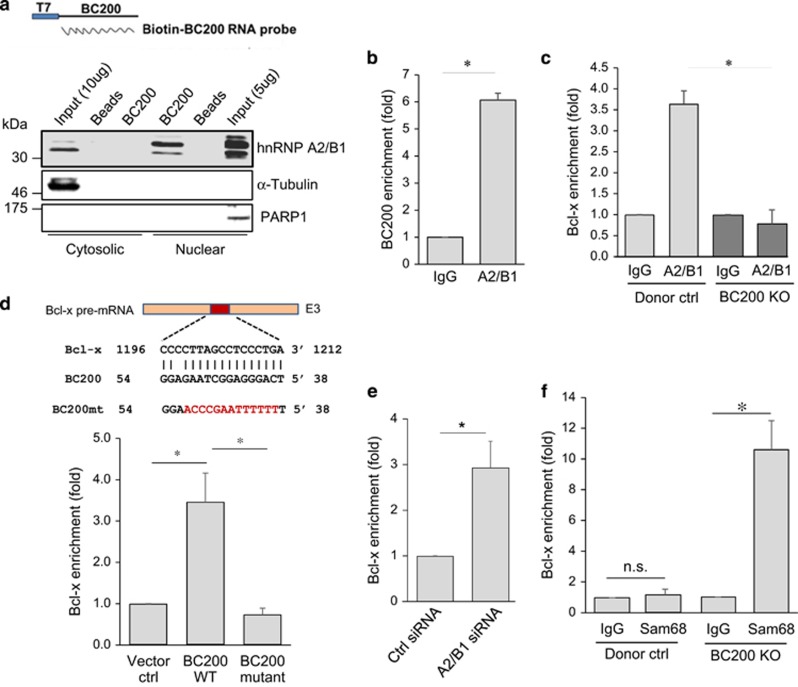
BC200 interacts with hnRNP A2/B1 and Bcl-x pre-mRNA. (**a**) Identification of hnRNP A2/B1 as a BC200-binding partner by RNA precipitation and western blot. A biotin-labeled BC200 RNA probe was used in pull-down experiments. PARP1 and *α*-tubulin serve as a nuclear marker and cytoplasmic marker, respectively. (**b**) Interaction of hnRNP A2/B1 with BC200, as determined by RIP assay using hnRNP A2/B1 antibody, followed by qRT-PCR. (**c**) BC200 is required for the interaction of hnRNP A2/B1 with Bcl-x pre-mRNA. Detection of interaction of hnRNP A2/B1 with Bcl-x pre-mRNA by RIP assay using hnRNP A2/B1 antibody, followed by qRT-PCR. (**d**) BC200 carries a 17-bp sequence complementary to Bcl-x exon 3. Also shown is the mutant sequence (top). The putative BC200-binding site in Bcl-x is essential to the interaction of hnRNP A2/B1 with Bcl-x pre-mRNA (bottom). BC200 KO#2 cells were transfected with vector control, BC200-WT or BC200-mutant. RIP assay was performed using hnRNP A2/B1 antibody 24 h after transfection. (**e**) Suppression of hnRNP A2/B1 increases the binding of Sam68 to Bcl-x pre-mRNA. RIP assay was performed using Sam68 antibody. (**f**) BC200 KO enhances the interaction of Sam68 with Bcl-x pre-mRNA, as detected by RIP assay using Sam68 antibody. Values are means±S.E. (*n*=3); *P<0.05

Next, we asked how BC200 KO promotes Bcl-xS. In this regard, several proteins have been implicated in regulation of alternative splicing of Bcl-x, including Sam68, ASF/SF2, heterogeneous nuclear ribonucleoprotein (hnRNP) A1, hnRNP A2/B1, hnRNP I and hnRNP K.^21, 22, 23, 24, 25, 26^ RNA precipitation using biotin-labeled BC200 RNA probe, followed by western blot, detected hnRNP A2/B1 (Figure 6a; Supplementary Figure S6) but not Sam68, ASF/SF2, hnRNP A1, hnRNP I or hnRNP K. The binding of hnRNP A2/B1 with BC200 was further confirmed by RNA immunoprecipitation (RIP) assay, which revealed about a sixfold enrichment of BC200 RNA with hnRNP A2/B1 antibody as compared with IgG control (Figure 6b). In contrast, little enrichment was seen for MALAT1 in the same RIP assay (Supplementary Figure S7A), suggesting that the interaction is specific to BC200. To determine the role of hnRNP A2/B1 in alternative splicing of Bcl-x, we knocked down hnRNP A2/B1 by RNAi (Supplementary Figures S5B and C). RT-PCR revealed that knockdown of hnRNP A2/B1 can increase Bcl-xS (Supplementary Figure S5D), confirming that hnRNP A2/B1 serves as a splicing silencer for Bcl-xS.

To determine whether BC200 is required for the binding of hnRNP A2/B1 to Bcl-x pre-mRNA, we performed RIP assay using hnRNP A2/B1 antibody. As expected, a 3.5-fold enrichment of Bcl-x pre-mRNA by hnRNP A2/B1 antibody was detected as compared with IgG in vector control cells (Figure 6c). However, the enrichment of Bcl-x pre-mRNA was lost in BC200 KO cells under the same experimental conditions (Figure 6c). This was not due to alterations in hnRNP A2/B1 expression, because BC200 KO had no effect on the expression level of hnRNP A2/B1, hnRNP A1, Sam68 or ASF/SF2 (Supplementary Figure S8).

Given the ability of hnRNP A2/B1 to regulate alternative splicing of many genes,^27, 28, 29^ we then determined whether BC200 is specific to the hnRNP A2/B1-mediated alternative splicing of Bcl-x. In the same BC200 KO cells, we were not able to detect any significant change in other known hnRNP A2/B1-regulated targets, including RON, CASP9, IRF-3 and A-Raf (Supplementary Figure S9). To further dissect the mechanism for this specificity, we found a 17-bp sequence in BC200 complementary with 3′-UTR of Bcl-x (exon 3) (Figure 6d; Supplementary Figures S7B and C). Therefore, we mutated this potential binding site and then conducted reconstitution experiments in BC200 KO cells using wild-type or mutant BC200. RIP assay using hnRNP A2/B1 antibody revealed that re-expression of wild-type BC200 in KO cells restored the ability of hnRNP A2/B1 to interact with Bcl-x pre-mRNA (Figure 6d). Most importantly, mutating the binding site (the 17-bp sequence) abolished the interaction of hnRNP A2/B1 with Bcl-x pre-mRNA (Figure 6d). Moreover, we found that the interaction of Sam68, a Bcl-xS promoting factor,^25, 26^ with Bcl-x pre-mRNA was significantly high when hnRNP A2/B1 was knocked down (Figure 6e), suggesting that hnRNP A2/B1 and Sam68 compete for Bcl-x pre-mRNA. Finally, we detected over a 10-fold enrichment of Bcl-x by Sam68 antibody in BC200 KO cells whereas no such enrichment was seen in control cells (Figure 6f), further suggesting that BC200 is required for hnRNP A2/B1 to compete with Sam68. Together, these results suggest that BC200 specifically facilitates the interaction of hnRNP A2/B1 with Bcl-x pre-mRNA, and at the same time this interaction prevents the binding of Bcl-x pre-mRNA to Sam68. Therefore, BC200 specifically determines the fate of Bcl-x splicing in this case.

## Discussion

It is now widely accepted that lncRNAs play a critical role in the regulation of various cellular processes and disease conditions ranging from pluripotency to cancer. Although BC200 is primarily expressed in neuronal cells, it is upregulated in various types of cancer, including breast cancer.^10, 11, 30^ However, the functional role of BC200 in breast cancer is poorly understood. Here, we present evidence that BC200 is an estrogen-regulated lncRNA capable of suppressing apoptosis by regulating alternative splicing of Bcl-x.

One of the interesting findings in this study is that BC200 expression is significantly higher in ER-positive tumors than in ER-negative tumors. A similar trend was also seen among ER-positive and ER-negative breast cancer cell lines. Further characterizations provide several lines of supporting evidence that BC200 is an ER-regulated lncRNA. First, BC200 can be induced by E2 and this induction can be suppressed by co-treatment with TAM, an ER antagonist. Second, there is a potential ERE located upstream of BC200 transcription. Third, luciferase assays with a reporter carrying the putative BC200 promoter or mutant ERE suggest that this ERE is critical to E2 regulation of BC200. Finally, ChIP assay using ER*α* antibody confirms the binding of ER to this ERE site in the BC200 promoter. However, ER is not the only factor responsible for BC200 expression because we also detect BC200 in ER-negative breast cancer cells.

Previous studies have shown that several lncRNAs in addition to BC200 are also regulated through ER signaling. For example, HOTAIR and Neat1 have been reported to be induced by E2.^31, 32^ In this regard, HOTAIR carries multiple functional EREs in its promoter region; in particular two of them reveal very strong activity in response to E2 in breast cancer cells.^32^ On the other hand, ER-regulated Neat1 serves as a critical modulator in prostate cancer.^31^ Therefore, identification of BC200 as an ER target gene increases the repertoire of ER-regulated lncRNAs. Like HOTAIR and Neat1, BC200 also plays an oncogenic role. However, the underlying mechanisms are fundamentally different. For example, HOTAIR regulates the chromatin state to promote cancer metastasis;^33^ similarly ER-induced Neat1 alters the epigenetic landscape of target gene promoters in prostate cancer.^31^ In contrast, BC200 promotes tumorigenesis by promoting expression of Bcl-xL. Crucially, it has been shown that increased expression Bcl-xL is associated with high risk of metastasis, reduced sensitivity to chemotherapeutic treatments and poor prognosis,^34, 35^ implying possible involvement of BC200 as an oncogenic lncRNA during breast cancer progression.

It is known that ER-regulated genes can play an important role in estrogen-driven tumor progression.^36^ In support of this notion, BC200 KO causes a significant change in the proliferative behaviors of breast cancer cells, as exemplified by their slow growth and increased propensity to undergo apoptosis. BC200 KO also causes cytochrome *c* translocation from mitochondria to cytosol, and PARP1 cleavage. Importantly, BC200 KO induces accumulation of pro-apoptotic Bcl-xS, providing a mechanism by which BC200 influences proliferation of breast cancer cells. Based on these findings, it is tempting to speculate that E2-induced BC200 might play an anti-apoptotic role during breast cancer progression.

Bcl-x is a member of the well-known Bcl-2 family that play key roles in apoptosis.^12^ This family include the anti-apoptotic members Bcl-2, Bcl-xL, Mcl-1, Bfl-1 and Bcl-w, and the pro-apoptotic members Bcl-xS, Bad, Bax, Bak, Bik, Bid, Bim, Puma and Noxa.^37, 38^ In particular, alternative splicing of Bcl-x can lead to expression of Bcl-xL or Bcl-xS with an opposite effect on cell apoptosis. Hence, alternative splicing plays a crucial role in the control of apoptosis. While Bcl-xL has an anti-apoptotic function and is often upregulated in several cancers, Bcl-xS is a pro-apoptotic protein that antagonizes the survival functions of Bcl-xL.^39^ Although the splicing change of Bcl-x is small between BC200 KO and BC200 intact cells (Figure 5), the ratio of Bcl-xS/xL could be critical. A delicate balance between Bcl-xL and Bcl-xS could determine the fate of cells, which may explain why the regulation of Bcl-x alternative splicing is complex. In this regard, several splicing factors such as SRSF1, hnRNP A1, hnRNP F/H and hnRNP I ^22, 25, 40, 41^ have been implicated in Bcl-x alternative splicing.

hnRNP proteins are a family of RNA-binding proteins that play multiple roles in the cell, including RNA processing, pre-mRNA splicing, mRNA export, localization, translation and stability.^42^ Like many other members of this family, hnRNP A2/B1 has been implicated in the regulation of alternative splicing of several genes, such as A-Raf, RON and IRF-3,^27, 28, 29^ in addition to Bcl-x. Thus, how does hnRNP A2/B1 manage to regulate a specific gene such as Bcl-x, but not others under the same condition? Our study provides a potential explanation. We suggest that hnRNP A2/B1 may serve as a basic machinery whereas BC200 may play a regulatory role given the requirement of BC200 for the binding of hnRNP A2/B1 to Bcl-x pre-mRNA. Through the binding site, BC200 interacts with Bcl-x pre-mRNA to form a complex containing hnRNP A2/B1, BC200 and Bcl-x pre-mRNA. Therefore, by manipulating the level or activity of hnRNP A2/B1, we would expect that all hnRNP A2/B1-regulated genes will be impacted. On the other hand, changing the level of BC200 would be able to specifically regulate Bcl-x splicing. This may explain why BC200 KO does not alter the splicing of other targets of hnRNP A2/B1. This mechanism may provide the cell with more flexibility to adapt to various environmental conditions.

In summary, BC200 is an ER-regulated target, raising the intriguing possibility that upregulation of BC200 in human breast tumors may play a critical role during breast cancer pathogenesis and progression by restraining apoptotic cell death owing to its ability to regulate Bcl-xL expression. Our study suggests that BC200 functions in partnerships with at least two players important for splicing (i.e., hnRNP A2/B1 and Sam68) to interact with Bcl-x pre-mRNA and regulate its alternative splicing (Supplementary Figure S10). In normal cells, the level of BC200 is low or undetectable.^10^ Thus, Sam68 is able to interact with Bcl-x pre-mRNA, leading to expression of Bcl-xS. In tumor cells, BC200 is increased such that it binds to Bcl-x pre-mRNA. This interaction facilitates recruitment of hnRNP A2/B1 to Bcl-x pre-mRNA to form a BC200-Bcl-x-hnRNP A2/B1 complex, which would suppress expression of Bcl-xS, but at the same time promote that of Bcl-xL. Consequently, these tumor cells become more proliferative and more resistant to anti-cancer therapy.

## Materials and Methods

### Cell culture

The human MCF-7, T47D and MDA-MB-231 cells were obtained from ATCC (Manassas, VA, USA). The MDA-MB-231 derivative cell lines (231 Br Met and BoM1883) were kindly provided by Dr Joan Massagué at Memorial Sloan-Kettering Cancer Center. Cells were cultured as described previously.^43^ For E2-deprivation experiments, cells were grown and maintained in phenol-red-free RPMI-1640 medium supplemented with 5% charcoal-stripped FBS, 2 mM l-glutamine, 100 U/ml penicillin and 100 *μ*g/ml streptomycin.

### KO of BC200 by CRISPR/Cas9

To facilitate the selection of positive clones, we generated a donor vector in such a way that targeting sequence is replaced by marker genes (GFP and PU (the puromycin resistance gene)) once it is integrated into the genomic DNA by HR. Donor vector carried ~800 bp targeting sequence at each side and EF1-GFP-T2A-PU in the middle, flanked by a LoxP site. The dual gRNA construct carrying Cas9 and donor vector were introduced into MCF-7 cells by transient transfection. As a control, everything was same except that the dual gRNA construct carried no BC200 specific sequences. One week later, the transfected cells were subject to puromycin selection; and surviving cells were sorted by FACS based on GFP signal into 96-well plates and then expanded as described previously.^16^

### TUNEL assay

Cells were washed twice with PBS, fixed with 4% paraformaldehyde for 15 min at room temperature and permeabilized in 0.25% Triton X-100 for 20 min at room temperature. TUNEL assays were performed according to the manufacturer's instructions (Invitrogen, Grand Island, NY, USA). Briefly, the cells were first incubated in TdT reaction cocktail for 60 min at 37 °C, followed by treatment with Click-iT reaction cocktail. The nucleus was stained with Hoechst 33342. For tumor tissues, TUNEL assay was performed using *in situ* cell death detection kit (Roche, Indianapolis, IN, USA) as per manufacturer's protocol. Briefly, paraffin-embedded tissues were deparaffinized and rehydrated, followed by treatment with proteinase K for 30 min at 37 °C. After PBS wash, TUNEL reaction mixture was applied on tissue samples and incubated for 60 min at 37 °C in the dark. The nucleus was stained with Hoechst 33342.

### Cytochrome *c* release assay

Cells were subject to immunostaining with anti-cytochrome *c* antibody as previously described.^44^ Briefly, cells were fixed in 4% formaldehyde, washed twice with cold PBS, permeabilized by 0.25% Triton X-100 in PBS for 15–20 min, blocked with goat serum and then incubated with cytochrome *c* antibody for 4 h at room temperature. Samples were washed twice with 0.2% Tween 20 in PBS and incubated with fluorescein–isothiocyanate-conjugated anti-mouse secondary antibody for 1 h at room temperature. DAPI staining of the cells was performed to visualize nuclear integrity.

### Immunohistochemistry

Immunohistochemistry (IHC) was used to detect Ki-67 in xenograft tumors using the procedure as previously described.^45^

### RNA precipitation

To identify potential BC200-binding partners, we performed the RNA precipitation assay using biotin-labeled BC200 RNA probe and then detected various proteins by western blot. DNA fragment covering the entire BC200 sequence was PCR amplified using a T7-containing primers and then cloned into pCR8 (Invitrogen). The resultant plasmid DNA was linearized with restriction enzyme *Not*I which was introduced from the reverse PCR primer, and then used to synthesize RNA by T7 polymerase. The remaining procedure was same as previously described.^46^

### RNA immunoprecipitation

To determine whether hnRNP A2/B1 interacts with BC200, we used hnRNP A2/B1 antibody to pull down hnRNP A2/B1 and then detected BC200 RNA by RT-PCR using BC200 specific primers. Magna RIP RNA-Binding Protein Immunoprecipitation Kit (Millipore, Billerica, MA, USA) was used for RIP procedures according to the manufacturer's protocol. After the antibody was recovered by protein A+G beads, standard RT-PCR was performed to detect BC200 RNA or Bcl-x mRNA in the precipitates. RIP with Sam68 antibody was carried out in the same way.

### Chromatin immunoprecipitation

ChIP assays were performed using a commercial kit from Millipore as per manufacturer's protocol. Briefly, cells were first fixed with 1% formaldehyde, and chromatin DNA was isolated and bound protein was digested with proteinase K. Quantitative PCR was performed using primers BC200-ChIP-5.1, BC200-ChIP-3.1 covering ERE site and BC200-ChIP-5.2, with BC200-ChIP-3.2 as negative control (see Supplementary Materials). Immunoglobulin G and non-specific antibody (anti-SUMO) were used as negative controls.

### Xenograft mouse model

Female nude (nu/nu) mice (4–5-week-old) were purchased from Harlan Laboratories (Indianapolis, IN, USA). All animal studies were conducted in accordance with NIH animal use guidelines and a protocol approved by the UMMC Animal Care Committee. MCF-7 cells with donor control and BC200 KO at the exponential stage were harvested and were then mixed with 50% matrigel (BD Biosciences, San Jose, CA, USA). Before tumor cell injection (1.5 million cells per spot), a 0.72 mg 17*β*-estradiol pellet (Innovative Research of America, Sarasota, FL, USA) was implanted beneath the back skin to facilitate tumor growth. Tumor cell injection was performed as described previously.^45^ Tumor growth was monitored, and tumor size was measured every other day. Tumor volume was calculated using the formula, volume=1/2 (length × width^2^). Paraffin-embedded tissues were prepared for IHC staining.

### Statistical analysis

Data are presented as mean±S.E.; the Student's *t*-test was used for assessing the difference between individual groups and *P*≤0.05 was considered statistically significant.

## Abbreviations

E2: estradiol
ER: estrogen receptor
EREs: estrogen response elements
hnRNP: heterogeneous nuclear ribonucleoprotein
IHC: immunohistochemistry
KO: knockout
lncRNA: long non-coding RNA
RIP: RNA immunoprecipitation
TAM: tamoxifen

## References

[bib1] Rinn JL, Chang HY. Genome regulation by long noncoding RNAs. Annu Rev Biochem 2012; 81: 145–166. 22663078 10.1146/annurev-biochem-051410-092902 PMC3858397

[bib2] Mercer TR, Dinger ME, Mattick JS. Long non-coding RNAs: insights into functions. Nat Rev Genet 2009; 10: 155–159. 19188922 10.1038/nrg2521

[bib3] Hu W, Alvarez-Dominguez JR, Lodish HF. Regulation of mammalian cell differentiation by long non-coding RNAs. EMBO Rep 2012; 13: 971–983. 23070366 10.1038/embor.2012.145 PMC3492712

[bib4] Prensner JR, Chinnaiyan AM. The emergence of lncRNAs in cancer biology. Cancer Discov 2011; 1: 391–407. 22096659 10.1158/2159-8290.CD-11-0209 PMC3215093

[bib5] Chen G, Wang Z, Wang D, Qiu C, Liu M, Chen X et al. LncRNADisease: a database for long-non-coding RNA-associated diseases. Nucleic Acids Res 2013; 41: D983–D986. 23175614 10.1093/nar/gks1099 PMC3531173

[bib6] Huarte M, Rinn JL. Large non-coding RNAs: missing links in cancer? Hum Mol Genet 2010; 19: R152–R161. 20729297 10.1093/hmg/ddq353 PMC2953740

[bib7] Martignetti JA, Brosius J. BC200 RNA: a neural RNA polymerase III product encoded by a monomeric Alu element. Proc Natl Acad Sci USA 1993; 90: 11563–11567. 8265590 10.1073/pnas.90.24.11563 PMC48024

[bib8] Kremerskothen J, Zopf D, Walter P, Cheng JG, Nettermann M, Niewerth U et al. Heterodimer SRP9/14 is an integral part of the neural BC200 RNP in primate brain. Neurosci Lett 1998; 245: 123–126. 9605471 10.1016/s0304-3940(98)00215-8

[bib9] Tiedge H, Chen W, Brosius J. Primary structure, neural-specific expression, and dendritic location of human BC200 RNA. J Neuroscie 1993; 13: 2382–2390. 10.1523/JNEUROSCI.13-06-02382.1993 PMC6576500 7684772

[bib10] Chen W, Bocker W, Brosius J, Tiedge H. Expression of neural BC200 RNA in human tumours. J Pathol 1997; 183: 345–351. 9422992 10.1002/(SICI)1096-9896(199711)183:3<345::AID-PATH930>3.0.CO;2-8

[bib11] Iacoangeli A, Lin Y, Morley EJ, Muslimov IA, Bianchi R, Reilly J et al. BC200 RNA in invasive and preinvasive breast cancer. Carcinogenesis 2004; 25: 2125–2133. 15240511 10.1093/carcin/bgh228

[bib12] Hetz C. BCL-2 protein family. Essential regulators of cell death. Preface. Adv *Exp Med Biol* 2010; 687: vii–viii. 20919634

[bib13] Shang Y, Hu X, DiRenzo J, Lazar MA, Brown M. Cofactor dynamics and sufficiency in estrogen receptor-regulated transcription. Cell 2000; 103: 843–852. 11136970 10.1016/s0092-8674(00)00188-4

[bib14] Wang C, Mayer JA, Mazumdar A, Fertuck K, Kim H, Brown M et al. Estrogen induces c-myc gene expression via an upstream enhancer activated by the estrogen receptor and the AP-1 transcription factor. Mol Endocrinol 2011; 25: 1527–1538. 21835891 10.1210/me.2011-1037 PMC3165912

[bib15] Sander JD, Joung JK. CRISPR-Cas systems for editing, regulating and targeting genomes. Nat Biotechnol 2014; 32: 347–355. 24584096 10.1038/nbt.2842 PMC4022601

[bib16] Ho TT, Zhou N, Huang J, Koirala P, Xu M, Fung R et al. Targeting non-coding RNAs with the CRISPR/Cas9 system in human cell lines. Nucleic Acids Res 2015; 43: e17. 25414344 10.1093/nar/gku1198 PMC4330338

[bib17] Boise LH, Gonzalez-Garcia M, Postema CE, Ding L, Lindsten T, Turka LA et al. bcl-x, a bcl-2-related gene that functions as a dominant regulator of apoptotic cell death. Cell 1993; 74: 597–608. 8358789 10.1016/0092-8674(93)90508-n

[bib18] Sumantran VN, Ealovega MW, Nunez G, Clarke MF, Wicha MS. Overexpression of Bcl-XS sensitizes MCF-7 cells to chemotherapy-induced apoptosis. Cancer Res 1995; 55: 2507–2510. 7780958

[bib19] Hossini AM, Eberle J, Fecker LF, Orfanos CE, Geilen CC. Conditional expression of exogenous Bcl-X(S) triggers apoptosis in human melanoma cells *in vitro* and delays growth of melanoma xenografts. FEBS Lett 2003; 553: 250–256. 14572633 10.1016/s0014-5793(03)01017-2

[bib20] Lindenboim L, Yuan J, Stein R. Bcl-xS and Bax induce different apoptotic pathways in PC12 cells. Oncogene 2000; 19: 1783–1793. 10777212 10.1038/sj.onc.1203495

[bib21] Revil T, Pelletier J, Toutant J, Cloutier A, Chabot B. Heterogeneous nuclear ribonucleoprotein K represses the production of pro-apoptotic Bcl-xS splice isoform. J Biol Chem 2009; 284: 21458–21467. 19520842 10.1074/jbc.M109.019711 PMC2755870

[bib22] Moore MJ, Wang Q, Kennedy CJ, Silver PA. An alternative splicing network links cell-cycle control to apoptosis. Cell 2010; 142: 625–636. 20705336 10.1016/j.cell.2010.07.019 PMC2924962

[bib23] Bielli P, Bordi M, Biasio VD, Sette C. Regulation of BCL-X splicing reveals a role for the polypyrimidine tract binding protein (PTBP1/hnRNP I) in alternative 5' splice site selection. Nucleic Acids Res 2014; 42: 12070–12081. 25294838 10.1093/nar/gku922 PMC4231771

[bib24] Chen ZY, Cai L, Zhu J, Chen M, Chen J, Li ZH et al. Fyn requires HnRNPA2B1 and Sam68 to synergistically regulate apoptosis in pancreatic cancer. Carcinogenesis 2011; 32: 1419–1426. 21642356 10.1093/carcin/bgr088

[bib25] Paronetto MP, Achsel T, Massiello A, Chalfant CE, Sette C. The RNA-binding protein Sam68 modulates the alternative splicing of Bcl-x. J Cell Biol 2007; 176: 929–939. 17371836 10.1083/jcb.200701005 PMC2064079

[bib26] Bielli P, Busa R, Di Stasi SM, Munoz MJ, Botti F, Kornblihtt AR et al. The transcription factor FBI-1 inhibits SAM68-mediated BCL-X alternative splicing and apoptosis. EMBO Rep 2014; 15: 419–427. 24514149 10.1002/embr.201338241 PMC3989673

[bib27] Golan-Gerstl R, Cohen M, Shilo A, Suh SS, Bakacs A, Coppola L et al. Splicing factor hnRNP A2/B1 regulates tumor suppressor gene splicing and is an oncogenic driver in glioblastoma. Cancer Res 2011; 71: 4464–4472. 21586613 10.1158/0008-5472.CAN-10-4410

[bib28] Ling H, Spizzo R, Atlasi Y, Nicoloso M, Shimizu M, Redis RS et al. CCAT2, a novel noncoding RNA mapping to 8q24, underlies metastatic progression and chromosomal instability in colon cancer. Genome Res 2013; 23: 1446–1461. 23796952 10.1101/gr.152942.112 PMC3759721

[bib29] Shilo A, Ben Hur V, Denichenko P, Stein I, Pikarsky E, Rauch J et al. Splicing factor hnRNP A2 activates the Ras-MAPK-ERK pathway by controlling A-Raf splicing in hepatocellular carcinoma development. RNA 2014; 20: 505–515. 24572810 10.1261/rna.042259.113 PMC3964912

[bib30] Hansji H, Leung EY, Baguley BC, Finlay GJ, Askarian-Amiri ME. Keeping abreast with long non-coding RNAs in mammary gland development and breast cancer. Front Genet 2014; 5: 379. 25400658 10.3389/fgene.2014.00379 PMC4215690

[bib31] Chakravarty D, Sboner A, Nair SS, Giannopoulou E, Li R, Hennig S et al. The oestrogen receptor alpha-regulated lncRNA NEAT1 is a critical modulator of prostate cancer. Nat Commun 2014; 5: 5383. 25415230 10.1038/ncomms6383 PMC4241506

[bib32] Bhan A, Hussain I, Ansari KI, Kasiri S, Bashyal A, Mandal SS. Antisense transcript long noncoding RNA (lncRNA) HOTAIR is transcriptionally induced by estradiol. J Mol Biol 2013; 425: 3707–3722. 23375982 10.1016/j.jmb.2013.01.022 PMC3679254

[bib33] Gupta RA, Shah N, Wang KC, Kim J, Horlings HM, Wong DJ et al. Long non-coding RNA HOTAIR reprograms chromatin state to promote cancer metastasis. Nature 2010; 464: 1071–1076. 20393566 10.1038/nature08975 PMC3049919

[bib34] Clarke MF, Apel IJ, Benedict MA, Eipers PG, Sumantran V, Gonzalez-Garcia M et al. A recombinant bcl-x s adenovirus selectively induces apoptosis in cancer cells but not in normal bone marrow cells. Proc Natl Acad Sci USA 1995; 92: 11024–11028. 7479929 10.1073/pnas.92.24.11024 PMC40563

[bib35] Olopade OI, Adeyanju MO, Safa AR, Hagos F, Mick R, Thompson CB et al. Overexpression of BCL-x protein in primary breast cancer is associated with high tumor grade and nodal metastases. Cancer J Sci Am 1997; 3: 230–237. 9263629

[bib36] Basu A, Rowan BG. Genes related to estrogen action in reproduction and breast cancer. Front Biosci 2005; 10: 2346–2372. 15970500 10.2741/1703

[bib37] Cory S, Adams JM. The Bcl2 family: regulators of the cellular life-or-death switch. Nat Rev Cancer 2002; 2: 647–656. 12209154 10.1038/nrc883

[bib38] Bouillet P, Strasser A. BH3-only proteins - evolutionarily conserved proapoptotic Bcl-2 family members essential for initiating programmed cell death. J Cell Sci 2002; 115: 1567–1574. 11950875 10.1242/jcs.115.8.1567

[bib39] Minn AJ, Boise LH, Thompson CB. Bcl-x(S) anatagonizes the protective effects of Bcl-x(L). J Biol Chem 1996; 271: 6306–6312. 8626425 10.1074/jbc.271.11.6306

[bib40] Garneau D, Revil T, Fisette JF, Chabot B. Heterogeneous nuclear ribonucleoprotein F/H proteins modulate the alternative splicing of the apoptotic mediator Bcl-x. J Biol Chem 2005; 280: 22641–22650. 15837790 10.1074/jbc.M501070200

[bib41] Bielli P, Bordi M, Di Biasio V, Sette C. Regulation of BCL-X splicing reveals a role for the polypyrimidine tract binding protein (PTBP1/hnRNP I) in alternative 5' splice site selection. Nucleic Acids Res 2014; 42: 12070–12081. 25294838 10.1093/nar/gku922 PMC4231771

[bib42] Han SP, Tang YH, Smith R. Functional diversity of the hnRNPs: past and present perspectives. Biochem J 2010; 430: 379–392. 20795951 10.1042/BJ20100396

[bib43] Singh R, Pochampally R, Watabe K, Lu Z, Mo YY. Exosome-mediated transfer of miR-10b promotes cell invasion in breast cancer. Mol Cancer 2014; 13: 256. 25428807 10.1186/1476-4598-13-256 PMC4258287

[bib44] Wu F, Chiocca S, Beck WT, Mo YY. Gam1-associated alterations of drug responsiveness through activation of apoptosis. Mol Cancer Ther 2007; 6: 1823–1830. 17575111 10.1158/1535-7163.MCT-06-0771

[bib45] Si ML, Zhu S, Wu H, Lu Z, Wu F, Mo YY. miR-21-mediated tumor growth. Oncogene 2007; 26: 2799–2803. 17072344 10.1038/sj.onc.1210083

[bib46] Zhang A, Zhou N, Huang J, Liu Q, Fukuda K, Ma D et al. The human long non-coding RNA-RoR is a p53 repressor in response to DNA damage. Cell Res 2013; 23: 340–350. 23208419 10.1038/cr.2012.164 PMC3587705

