# Supplementary material for: Regulation of alternative splicing of Bcl-x by BC200 contributes to breast cancer pathogenesis
Source: Cell Death Dis. 2016 Jun 9;7(6):e2262–. doi: 10.1038/cddis.2016.168 (PMC5143396; doi:10.1038/cddis.2016.168)
Supplement: Supplementary Information [file cddis2016168x1.pdf]

Regulation of alternative splicing of Bcl-x by BC200 contributes to breast cancer pathogenesis

Singh et al

Supplementary materials and methods

Reagents

Primary antibodies were obtained as follows: PARP-1 and cytochrome c from Cell Signaling Technology (Beverly, MA), Bcl-x, hnRNP A2/B1, hnRNP A1, ASF/SF2, Sam68 and Bcl-2 from Santa Cruz Biotechnology (Dallas, TX); Ki-67, GAPDH from ProteinTech (Chicago, IL). Secondary antibodies conjugated with IRDye 800CW or IRDye 680 were purchased from LI-COR Biosciences (Lincoln, NE). siRNAs against hnRNP A2/B1 were obtained from Santa Cruz Biotechnology. PCR primers were purchased from IDT (Coralville, IA). Breast cancer tissue cDNA arrays were purchased from OriGene (Rockville, MD).

Expression vectors

The high fidelity enzyme Phusion (ThermoFisher) was used to amplify respective DNA fragments by PCR to make these constructs. Bcl-xS expression vector was constructed by first PCR amplification using primers Bcl-xS-R1-Myc-5.1 and Bcl-xS-Not1-Myc-3.1 (Table S1), and then cloned into pCDH-CMV-copGFP carrying Myc tag at EcoR I and Not I sites by cold fusion kit as described previously (1). BC200 was cloned into pCDH-MSCV-copGFP using the same approach and it was amplified using primers BC200-MSCV-R1-5.1 and BC200-Not1-3.1. To generate mutant BC200 at potential bindings for Bcl-x, we used two step PCR approach. We first performed PCR using primers BC200-MSCV-R1-5.1A and BC200-Not1-3.1. We then used the first PCR product as a template and performed second round of PCR using primers BC200-MSCV-R1-5.1B and BC200-Not1-3.1. To clone the putative BC200 promoter, we PCR amplified a 2 kb fragment from human genomic DNA using primers Luc-BC200p-Kpn1-5.1 and Luc-BC200p-Xho1-3.1 and then directly cloned into pGL3 vector at Kpn I and Xho I sites. Mutant promoter was generated by PCR using primers Luc-BC200p-Kpn1-5.1 and BC200p-m1-3.1; BC200p-m1-5.1 and Luc-BC200p-Xho1-3.1. The two PCR products were directly cloned into pGL3 basic vector at Kpn I and Xho I sites using Cold Fusion kit. To make BC200 RNA probe, we amplified BC200 using

primers T7-BC200-5.1 and BC200-Not1-3.1and then cloned into PCR8 using TA cloning kit (Life Technology). The sequences of all PCR products were verified by DNA sequencing.

Transfection

Cells were transfected with siRNAs using RNAfectin reagent (Applied Biological Materials, Vancouver, Canada) or plasmid DNA using DNAfectin (Applied Biological Materials) following the manufacturer's protocol as previously described (1)

MTT assay

MTT assay was performed to determine the effect of BC200 knockout (KO) on cell growth as described previously (3). In brief, vector control or BC200 KO cells were seeded in 96-well plates and cultivated for 4 days before subjecting to MTT assays.

RNA preparation and RT-PCR

For RT-PCR, we isolated total RNA using Trizol reagent (Invitrogen, Carlsbad, CA) as per the manufacturer's protocol and used 0.5 μg RNA to synthesize cDNA by SuperScriptase III (Invitrogen) with random primers. Finally, the resultant cDNA was used for PCR reactions. PCR annealing temperature varied depending on the primers used, followed by SYBR Green qPCR as described previously (4). GAPDH or β-Actin were used as an internal control.

Luciferase assay

MCF7 cells were grown in E2 free medium in 12-well and transfected with 400 ng of BC200 promoter reporter constructs along with 2 ng of pRL-SV40 renilla luciferase construct as an internal transfection control. Control transfections were performed using pGL3 basic vector alone. Twenty-four h after transfection, the cells were treated with 1 nM E2, incubated for an additional 18 h, and then harvested for luciferase assays using dual-Glo Luciferase Assay kit (Promega). Renilla luciferase was used for normalization.

Western blotting

Cells were harvested and protein was extracted from cells as previously described (2). The protein concentration was determined using a protein assay kit (Bio-Rad, Hercules, CA, USA) and

samples were separated in SDS polyacrylamide gels, with various concentrations depending on the molecular weight of the protein under investigation. After probing with a primary antibody, the membrane was incubated with a secondary antibody labeled with either IRDye 800CW or IRDye 680. Finally, signal intensity was determined using the Odyssey Infrared Imaging System (LI-COR Biosciences, Lincoln, NE, USA).

Primers used in this study

Bcl-X-RT-5.1 ATGGCAGCAGTAAAGCAAGCG

Bcl-X-RT-3.1 TCATTTCCGACTGAAGAGTGA

Bcl-X-RT-5.2 GAGGCAGGCGACGAGTTTGAA

Bcl-X-RT-3.2 TGGGAGGGTAGAGTGGATGGT

BCL-XL-F TAAACTGGGGTCGCATTGTG
BCL-XL-R AGGTAAGTGGCCATCCAAGC
BCL-XS-F GCAGTAAAGCAAGCGCTGAG
BCL-XS-R GTTCCACAAAAGTATCCTGTTCAAAG
Bcl-xS-R1-Myc-5.1 CCATGGAGGCCCGAATTCTGTCTCAGAGCAACCGGGAG

Bcl-xS-Not1-Myc-3.1 TCGCAGATCCTTGCGGCCGCTCATTTCCGACTGAAGAGTGA

BC200-MSCV-R1-5.1 TTCTAGAGCTAGCGAATTCGGCCGGGCGCGGTGGCTCAC
BC200-Not1-3.1 TCGCAGATCCTTGCGGCCGCAAAGGGGGGGGGGGTTGTT
BC200-MSCV-R1-5.1A
TTCTAGAGCTAGCGAATTCGGCCGGGCGCGGTGGCTCACGCCTGTAATCCCAGCT
BC200-MSCV-R1-5.1B
CACGCCTGTAATCCCAGCTCTTTTTTTAAGCCCAAGGCGGGAGGATAGCTTGAGCC
C

BC200-T1 ATAACCCTATGGCCAGCAGA

BC200-T2 TTAAGAAGCTGAGGAAAGCA

CHIP-BC200-5.1 AGTGGTGCTGTCTCGGCTCA
CHIP-BC200-3.1 GCGCAGTGGCTCATGCCTGT

CHIP-BC200-5.2	TACGGAGTAATCCTTGAGCA
CHIP-BC200-3.2	AGAGTTTCCGTGTGACTTTT
A-Raf-RT-F1	AAATCTAAGGCTCCATGG
A-Raf-RT-R1	CCCGGACAGTCACTCACCA
A-Raf-RT-R2	CCATTTCGCTTCCATGAC
IRF-3-RT-F	AGCCTCGAGTTTGAGAGCTACC
IRF-3-RT-R	GGTATCAGAAGTACTGCCTCCAC
RON-F	TGTGAGAGGCAGCTTCCAGCAG
RON-R	CTAGCTGCTTCCTCCGCCAC
CASP9-F	AGACCAGTGGACATTGGTTC
CASP9-R	GGTCCCTCCAGGAAACAAA
BC200p-m1-5.1	AAACTCCATATATCACCGATTCCGCCCACCTCGGCCTCC
BC200p-m1-3.1	TGGGCGGAATCGGTGATATATGGAGTTTCGAGACCTCCCT
Luc-BC200p-Kpn1-5.1	TTTCTCTATCGATAGGTACCTACGGAGTAATCCTTGAGCA
Luc-BC200p-Xho1-3.1	CTTAGATCGCAGATCTCGAGTCTTATTCTCGATTGAAATT
T7-BC200-5.1	TAATACGACTCACTATAG GGGGCCGGGCGCGGTGGCTCAC
BC200-RT-5.2	TCACGCCTGTAATCCCAGCT
BC200-m-RT-5.1	TTTTTTTAAGCCCAAGGCGG
BC200-right-R1-5.2	TTATACGAAGTTATGAATTCTGGGAGAAGTTAAAGCCATT
BC200-left-BamH1-3.2	GCTATACGAAGTAGGGATCCATCAAAAACAAGGATGACTC

Reference

1. Singh R, Pochampally R, Watabe K, Lu Z, Mo YY. Exosome-mediated transfer of miR-10b promotes cell invasion in breast cancer. Molecular cancer. 2014;13:256.
2. Sachdeva M, Zhu S, Wu F, Wu H, Walia V, Kumar S, et al. p53 represses c-Myc through induction of the tumor suppressor miR-145. Proceedings of the National Academy of Sciences of the United States of America. 2009;106:3207-12.
3. Wu F, Chiocca S, Beck WT, Mo YY. Gam1-associated alterations of drug responsiveness through activation of apoptosis. Molecular cancer therapeutics. 2007;6:1823-30.
4. Si ML, Zhu S, Wu H, Lu Z, Wu F, Mo YY. miR-21-mediated tumor growth. Oncogene. 2007;26:2799-803.

Fig S1

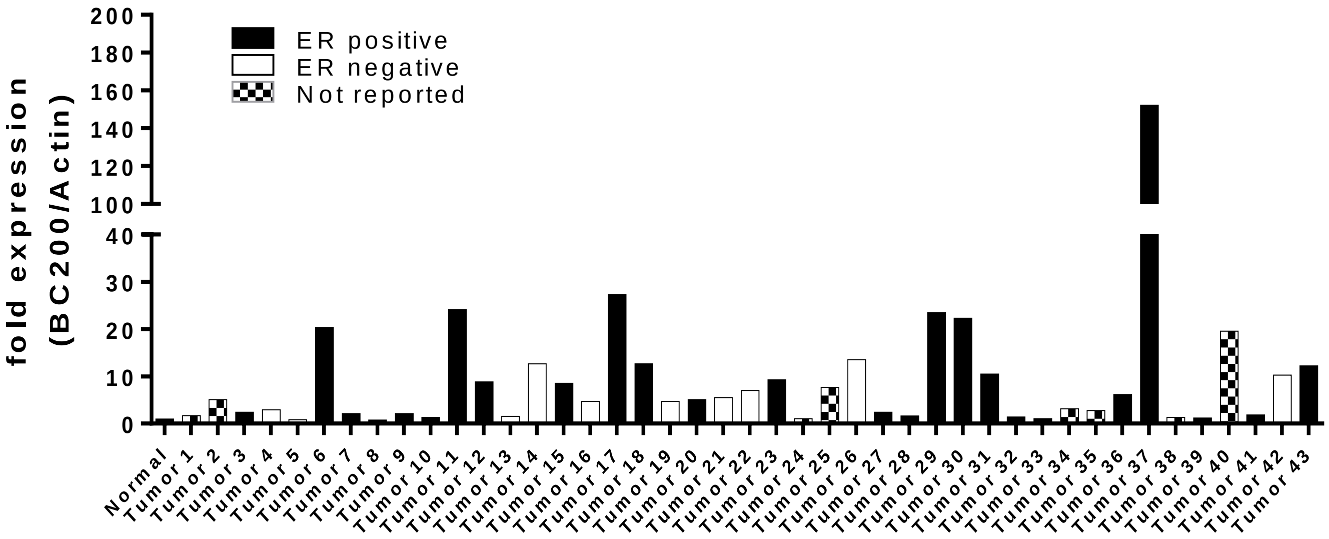

Fig S1. RT-qPCR analysis of breast cancer tissue scan. Five normal and 43 breast cancer tissue of different grades and estrogen status were analyzed by RT-qPCR (Data shown as fold change in lncRNA BC200 level against average value of 5 normal samples as 1).

Fig S2

A

```
TACGGAGTAATCCTTGAGCATCCCCTGACTCAGAGCACTGCCACCGCATCAGCTTCTTCCAGATGC
CAAGAAGCACCTCTGGGTCTTGGGGTTCCGGCTTGTTTGTGTCACAAGTAATAACTTGTATTTTAT
TTGCTTTTAAAAACTGGATATGCTCCCACTTCTCAGCCTCTACAATGAGACCTGTGTTTCGTGTTT
TTCAGTGTGCCTTTGAGAAAGGCCCATAAACCTATAAACACCCTTTTTCTCCCCAAGGTGAGGTTT
GACCCTAGTACTTACTACCACTGTTTACTGGAGAGTCATGTTGAGAACATCTCCTTTAGGCTCTAT
TTCTCATCCTAAGGGATATATCCAGCCTTCATTATGGAAGGTTTTGAAGGCCTCAAATAAAATCAA
AGTAGAATTTGAATAAAACATCTTGGCTGGGCACGGTGGCTCGCATCTGTAATCCCAGCACTTTGG
GAGGCTGAGGTGGGCGGGTCACAAGGTCAGGAGATCAAGAACATCCTGGCTAACACGGTGAAACCC
CCATCTCTGCTAAAAATACAAAAATCAGCTGGGCGTGGTGGCGCGTGCCTGTAGTCCCAGCTACTC
GGGAGGCTGAGGCAGGAGAATCGCTTGAACCAGGGAGTCGGAGGTTGCAGTGAACCGAGATCACAC
CACTGCACTCCAGCCTGGCGACAGAGAGACTCTGTCTCAAATAAATAAATAAAACATCTTAAAGAA
ATAGAAAATACTAGTAGAGTATTCACTCTGATTTCAGAATCAATTTGTGGAAATTCAGTTTCACAA
GAATGAAAAAAGCCCCCTAAGCAAGGTAGCTTTTTTTTTTTTTTTTAAAGATGGGGTCTCACTATG
TTTTCCAGACTGATCTCAAACTCCTGGGCTCAAGCAATCCTCCTCCCTCAGCCTCCTGAGTGGCTG
GGACTACAGGTACACATCACCACCACCCTGCTAGGATATGGTAGCTTTTTAACCAAAACACACCAC
CATGCAATGGAGAGCATGTAACTTGTTATTTGGTCATACAAATACATCAGAATAAATTCCACCTCT
CTTGTGGAGCCCTTTTTCCTACATCTGCACAGAAACTGGCACTAGCAGGACTGCAACCGAGGCAGA
CTCGAGAATTAGCAGACTCTGGCTCAGGCAGGACAATAGAGCAGCGGGAAATTTCTTTCAAGGGCT
TTTGTTGCTGTTGTTGAGATGGAATCTTGCTGTGTCACCCAGGCTGGAGTGCAGTGGTGCTGTCTC
GGCTCACTGCAATCTCCGCCTCCCAGGTTTAAACGATTCTCCTGCCTCAGCCTCTCAAGTAGCTGC
GATTACAGGCATGTGCCACCATGCCCAGCTAATTTTTGCATTTTTGGTAGAGATGGGGTTTCACTA
TGTTGCTCAGGGAGGTCTCGAAACTCCTGACCTCAGGTGATCCGCCCACCTCGGCCTCCCAAAGTG
TTGAGATTACAGGCATGAGCCACTGCGCCCGGCTTGAGACCTTGAGGGGCTTTTTAACCAAGAGAT
GGTGTAGGGACAGAAGCCAGCAAGAAGAACATGGTGGACTCTCTGTGAGGCCCCAAACCCCAAATC
ATGAGCCTCCGCCCCCTGAAAGTATTCCAGGATCTCTGAGGGAGGTTTTGGGAGGGGGCCAAGGTC
CTGCAATTGGGTGGGTGGGGCTCCAAGCCATTGTTCTTTGCACATTCATGCCAATGTGACCTAATT
GGCTAAGCACAGGCTGGAGAAGCTAGATAAACCTATTTAAATTCAGAGCCAAAAGACACTCAAGGA
TGTTTTCTGAGGGGGTGTGAGGGTTGGAAAATCATCCTATATTTGATTTATCTCCCACCTCATGTC
CTGAGTCAATTCCTTAGTCTTAGATGAGGGAGACGACTTGGGAGTCATCCTTGTTTTTGATGAGCT
ATATAACCCTATGGCCAGCAGAGGGAAGTACTGCATTTCAGAGCGACAATTTGAGATCTATGAAAG
AATTTCAATCGAGAATAAGA
```

B

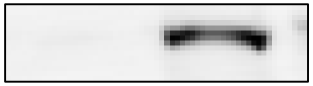

Fig S2. A, 2 kb DNA sequence upstream of BC200 transcription. The putative ERE is highlighted in blue. B, ER pulldown followed by Western blot (ERα).

Fig S3

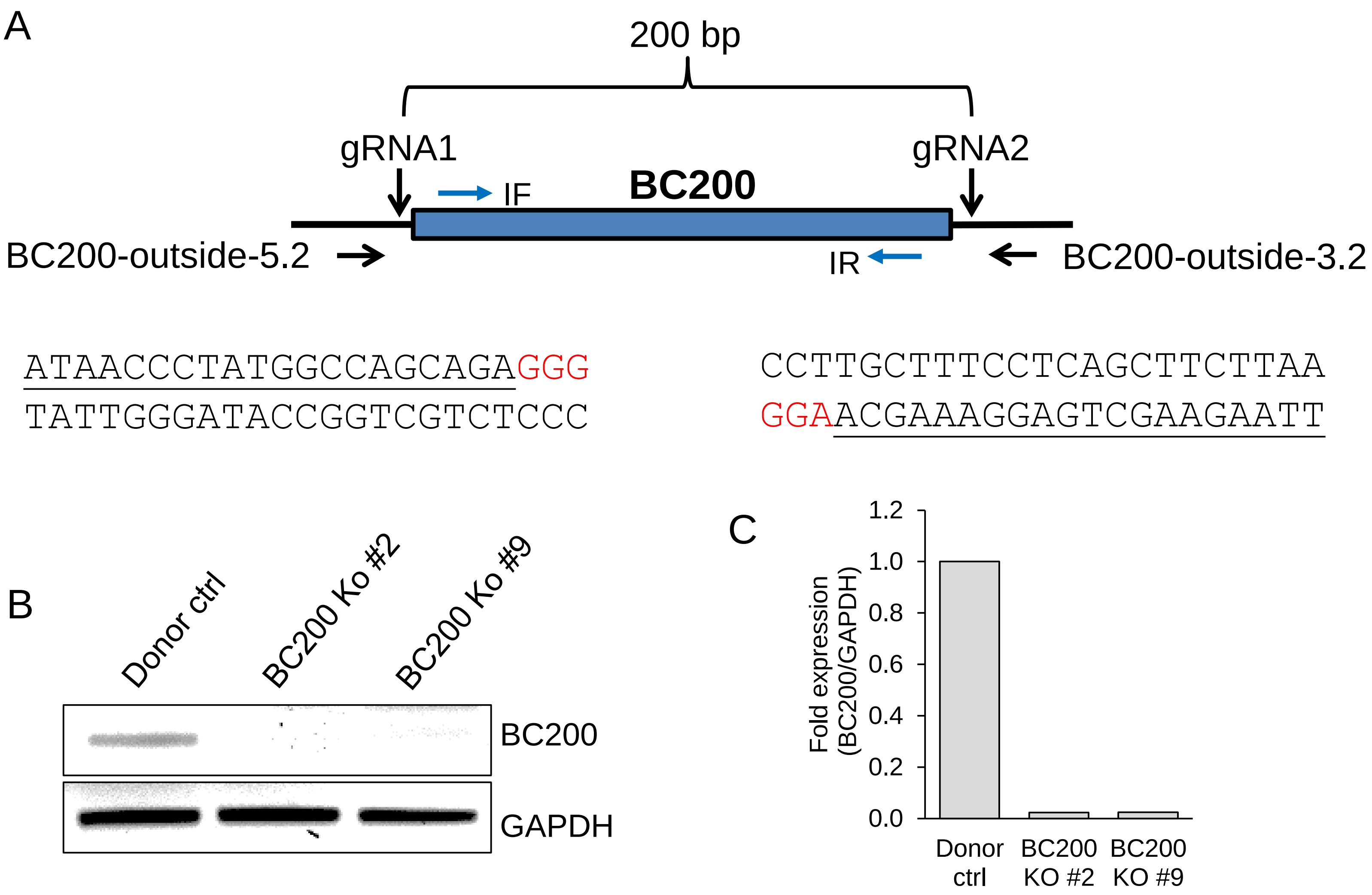

Fig S3 Knockout of BC200 by CRISPR/Cas9. A, Relative location of two gRNAs outside of BC200 with sequences indicated below. B, Identification of BC200 knockout by genomic PCR using primers inside forward (IF) and inside reverse (IR). C, RT-qPCR analysis of BC200 level in donor control and two knockout clones.

Fig S4

A

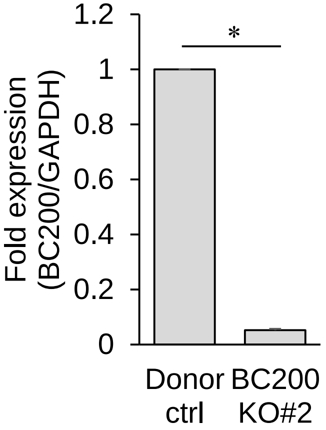

B

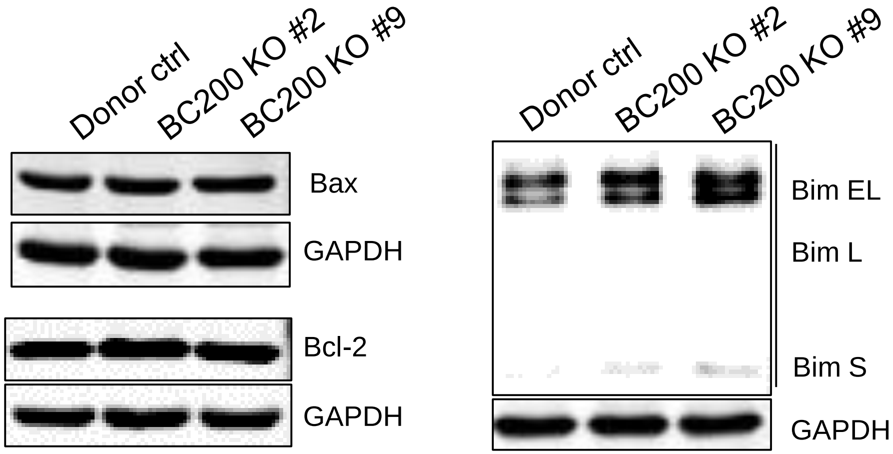

Fig S4. A, Detection of BC200 by qRT-PCR for tumor samples (a mixture of 3 samples). B, Effect of BC200 knockout on expression of Bcl-2, Bax and Bim as detected by Western blot. A slight increase in the Bim level was detected in BC200 knockout clones**.**

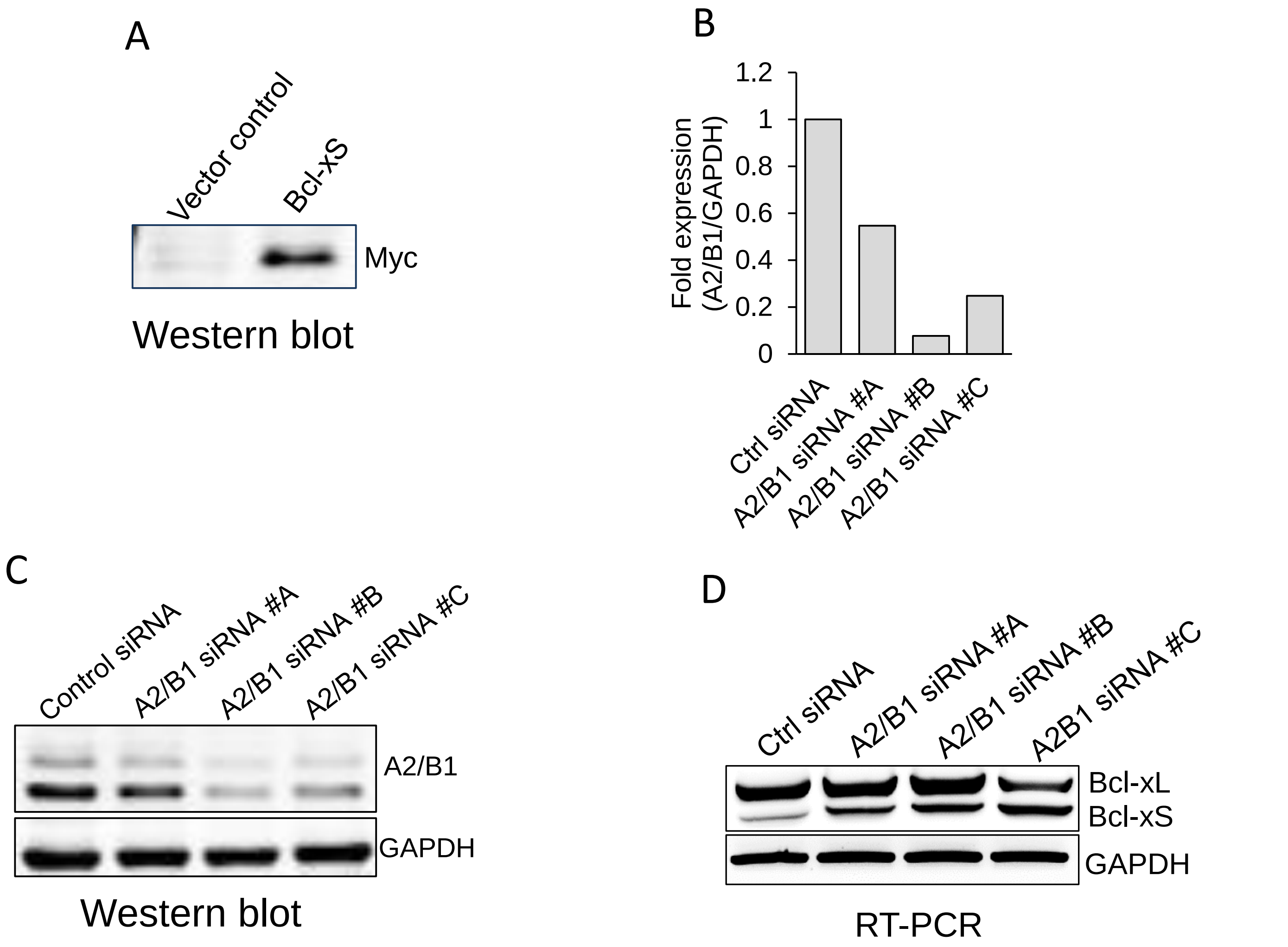

Fig S5. Silencing of hnRNP A2/B1 promotes Bcl-xS. A, Confirmation of Bcl-xS expression by Western blot. Bcl-xS was cloned into pCDH-myc-tag vector and transient transfection was done in MCF-7 cells. Myc-tag antibody was use to confirm Bcl-xS expression. B, Detection of hnRNP A2/B1 cells by qRT-PCR after transfection of hnRNP A2/B1 siRNAs in MCF-7. C, Western blot to confirm the knockdown of hnRNP A2/B1 24 h after siRNA transfection. D, hnRNP A2/B1 knockdown by RNAi induces expression of pro-apoptotic Bcl-xS isoform, as detected by RT-PCR.

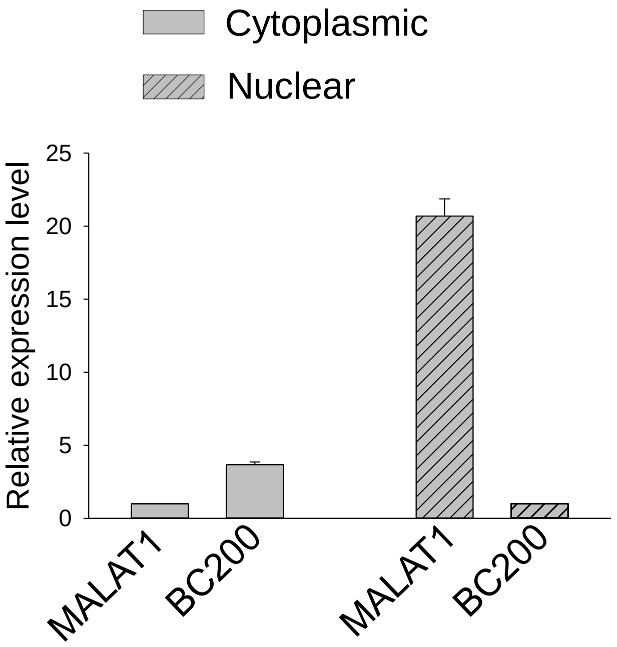

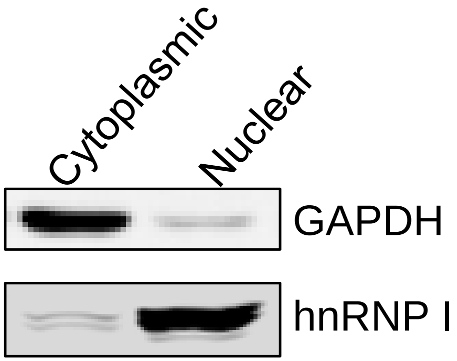

Fig. S6 Detection of BC200 in cytoplasm and nucleus. MALAT1 serves a control. Left, qRT-PCR results; right, cytoplasmic and nuclear fractions as detected by Western blot.

Fig. S7

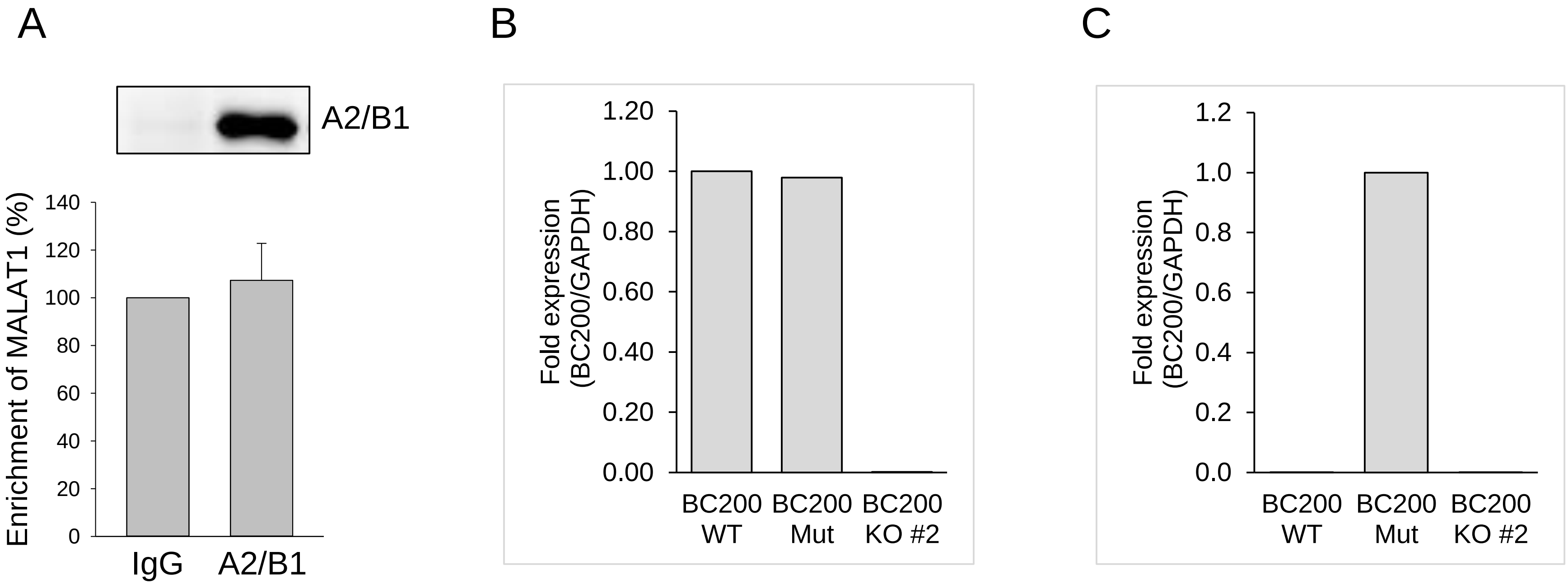

Fig. S7 A, Detection of hnRNP A2/B1 and MALAT1 by RIP with hnRNP A2/B1 antibody. B, Detection of WT BC200 and mutant BC200 by qRT-PCR using common primers after re-expression in the KO cells. C, Detection of mutant BC200 by qRT-PCR using mutant specific primers after re-expression in the KO cells.

Fig. S8

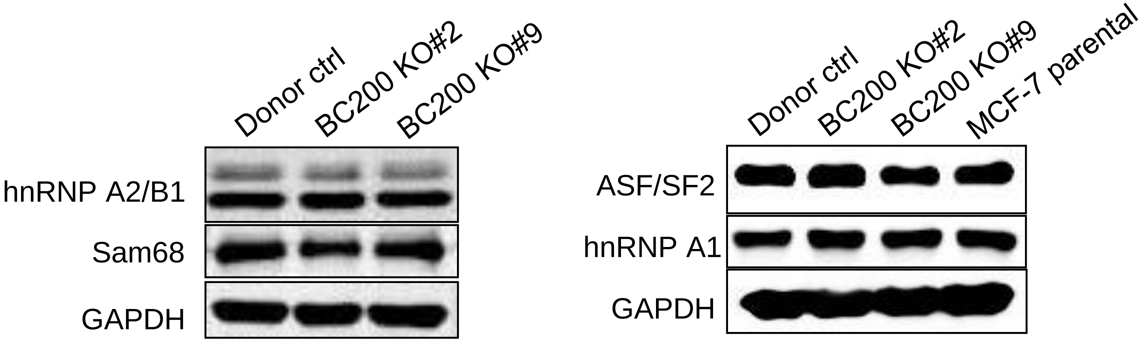

Fig S8 Detection of hnRNP A2/B1, Sam68, ASF/SF2 and hnRNP A1 in BC200 KO and control cells. BC200 KO has no effect on expression level of hnRNP A2/B1, ASF/SF2, hnRNP A1 or Sam68 which have been previously shown to be involved in the alternative splicing of Bcl-x.

Fig. S9

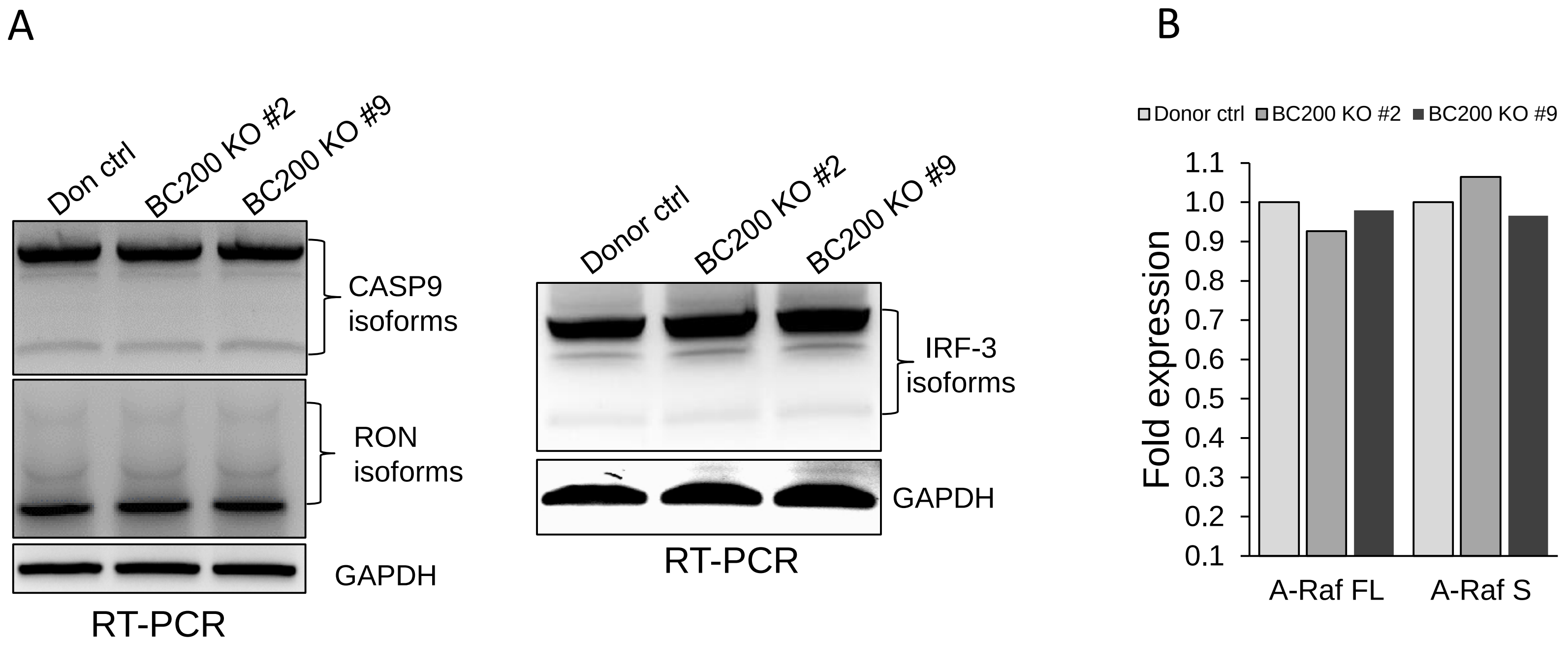

Fig S9 BC200 KO does not affect the alternative splicing of other targets of hnRNP A2/B1 such as RON, CASP9 and IRF-3, and A-Raf. A, RT-PCR was performed using specific primers that cover all splice variants of RON, CASP9 and IRF-3 in control and BC200 KO cells. B, Detection of full-length (FL) or a short form (S) A-Raf by qRT-PCR. RNA was extracted from donor control or BC200 KO cells. Primers for FL are A-Raf-RT-F1 and A-Raf-RT-R1; primers for S are A-Raf-RT-F1 and A-Raf-RT-R2.

Fig. S10

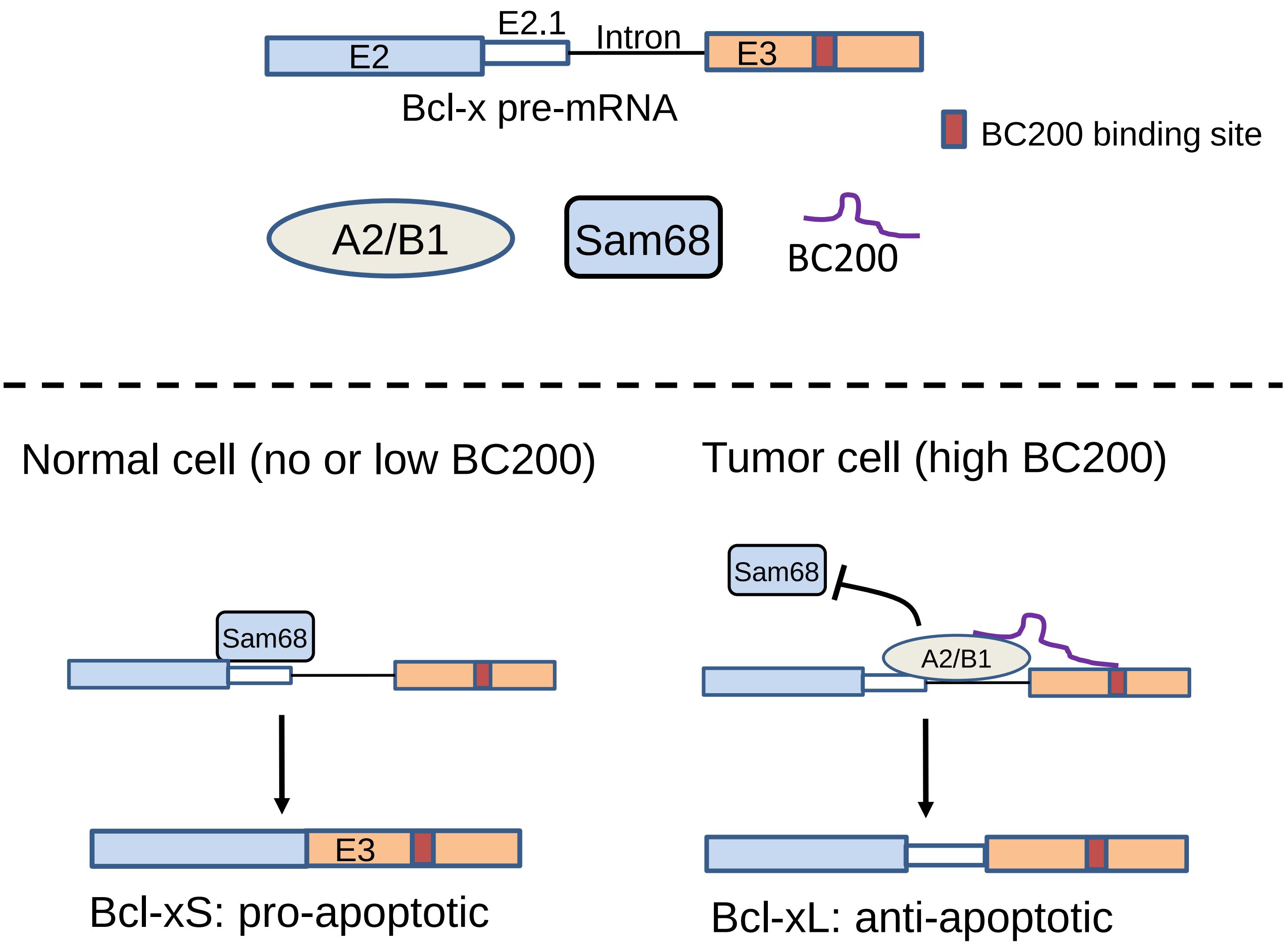

Fig S10 A working model for BC200-mediated expression of Bcl-xL. See text for details.
